# A Treatise on Retention of Urine, Caused by Strictures in the Urethra, &c.

**Published:** 1832-07-01

**Authors:** 


A Treatise on Retention of Urine, caused by Strictures in the Urethra; and on the Means by which Obstructions of this Canal may be effectually removed. By *Theodore Ducamp,* Doctor of Medicine of the Faculty of Paris, &c. &c. Translated from the French, with Notes and Additions, by *William M. Herbert,* M.D. Octavo, pp. 219—five Plates. New York, 1827.

We believe we may assert, without fear of contradiction from those who are well informed upon the subject, that the diseases of the urinary organs are at present better understood and better treated in this, than in any other country. The works of Hunter, Home, Wilson, Bell, and the recent papers of Brodie, in the surgical department, with the modern chemical investigations of foreign, as well as British chemists and physicians, and last, not least, the labours of Dr. Prout, have increased our information and improved our practice in a degree almost unprecedented, in so short a space of time and in so difficult an investigation. It is generally imagined that our treatment of strictures is superior to that of the same disease by the French surgeons; and from what we have read, as well as from what we have heard, we imagine that the preference is a just one. The employment of force and violence is too much abused by the French, and one great object of the work we have now before us is to discountenance this very abuse. The following quotation, from Deschamps, gives an instance of violence on the part of one of the ornaments of Gallic surgery.

"Desault, who had the reputation of the greatest skill and dexterity in the introduction of the catheter, and who has chiefly emboldened surgeons by his example to employ force for this purpose, deviated, like others, and even very widely, from the axis of the canal. The following fact is a remarkable proof of it: it is related by M. Deschamps, to whom it was communicated by M. Garre. Being called in the year 1795 to a patient who had great difficulty in making water, he (M. Garre) was much surprised to find him make use of two chamber pots, the one to receive the urine which flowed by the usual course, and the other to catch that which was voided by the anus. He learned from the patient, that in an attack of retention of urine, which he had suffered, Desault found great difficulty in passing the catheter, and therefore had had recourse to force, by which he succeeded in evacuating the bladder. There can be no doubt that in this case, the point of the instrument had perforated the mucous membrane of the urethra and the rectum, and afterwards passed through the intestine, again to enter the bladder above the prostate. Cases like this would appear very interesting to compare with the precept above inculcated—but unfortunately, these are not the cases which usually embellish our journals. (Deschamps.) Traité historique et dogmatique de l'operation de la taille. Vol. I. p. 238." 63.

Our object is not to analyze this treatise, nor even to offer a formal critique upon it. The author proposes a new plan of treating strictures, and we shall merely present this to our readers as briefly as we are able. Their treatment he considers as essentially founded on a two-fold indication: *to destroy the morbid disposition of the parts which form the stricture, and to re-*

*duce them to a level with the rest of the canal.* In order to effect the destruction of the stricture, the author recommends caustic as the most efficient means, and the dangers and inconveniences which have hitherto occasionally followed its employment, have been owing, he thinks, to the manner of using it. The caustic should come in contact with nothing but the stricture, but should touch it, from within outwards, throughout its whole extent. For instance, if the stricture should be seated at the upper part, the caustic should be applied to that only, and so on. It must be obviously necessary to ascertain the exact site of the stricture, previous to following this recommendation.

"It is important to determine with precision at what distance from the orifice of the glans, the stricture we propose to treat is situated. For this purpose the canal must be explored with a catheter of middle size, in order that it may pass readily through the sound parts of the urethra, and be arrested by the first strictured point that it encounters. It is customary to employ a plaster bougie on which a mark is made with the nail near the orifice of the glans. I make use of a hollow bougie of gum-elastic, of the size No. 6, on which are marked the divisions of a foot rule. On introducing this bougie, I always know the distance to which it has penetrated the urethra; and when it is stopped by a stricture I perceive immediately that such stricture is at the distance of so many inches and lines from the orifice, which I note down.

Having ascertained this point, I immediately proceed to the examination of another, which is the situation of the opening in the stricture. In order to do this, I take an impression of the stricture in wax, and I obtain in relief the form of its interior extremity. For this purpose I make use of the following instrument, which I call an *exploring catheter.* I have catheters of the Nos. 8, 9, and 10, open at both ends, and marked with the divisions of the foot; the anterior opening of these instruments must be about half the size of the other; I take a bit of sewing silk, and having tied several knots in it, and dipped them in melted wax, I round off the wax in the manner represented in fig. 7, plate I. By means of a bit of edging I pass the silk into the catheter, entering at the larger opening; when it has reached the other opening, the bulb formed by the knots covered with wax is detained, while the silk passes on and forms at the extremity of the catheter, a pencil of fine downy threads, both soft and strong; or else I pass the bit of flat silk through four little holes situated near the end of the instrument, and uniting them together in a knot, I afterwards spread them out in the form of a pencil. This pencil is soaked in a mixture composed of equal parts of yellow wax, diachylon, shoe-maker's wax, and resin; I take a sufficient quantity of it to enable it when rounded off to equal the bulk of the catheter. I let this *moulding wax* grow cold, and softening it between my fingers, roll it upon some hard polished surface. I cut this kind of bougie added to the gum-elastic canula, at about two lines from the extremity of the latter, and round off the wax like the end of a catheter. By this arrangement, the moulding wax, mingled with the filaments of silk, becomes incorporated with them, and cannot fall off. I introduce one of these catheters into the urethra, and after I have arrived at the stricture, I leave the instrument in its place for a few moments, in order that the wax may have time to grow warm and soften, when the catheter is pushed forward; the wax being thus *pressed* between the catheter and the stricture, fills all the sinuosities of the latter, enters its opening, and, in a word, is moulded according to its figure. The catheter being completely withdrawn, I find at its extremity the form of the stricture.

If the projection of wax which has entered the stricture, be in the middle of the lump of the same material which terminates the catheter, I perceive that the projecting parts which constitute the stricture, are equally distributed around the opening, and that the whole circumference of the latter is to be cauterized. But if it be at the upper part that the projection to be destroyed is situated, at the lower side; if, on the contrary, it be at the inferior part, I know that the caustic must be directed to the upper portion, and in like manner of the sides. By this means I can always procure the shape of the stricture, and ascertain all the changes that it undergoes in the course of the treatment. In a word, I can ascertain as clearly what takes place in the stricture at the deepest part of the canal, as if it were exposed to my sight." 107.

Some precautions are necessary in the use of this "exploring catheter," but for these we must refer to the book itself. In order to determine the length of the stricture, that is, its extent from before backwards, Dr. Ducamp has small cylindrical bougies of gum-elastic, which he covers with moulding wax, by taking several filaments of flat silk, soaking them in the wax melted, then winding this silk thickly coated with wax round the bougie, and rolling the latter between two polished bodies. This bougie is introduced, suffered to remain in the stricture a few moments, and when withdrawn it bears a furrow, the extent of which denotes that of the stricture. But this plan can only answer in cases where the bougie will enter the stricture; in some it will not, and then M. Ducamp has another expedient.

"Having taken an impression of the stricture, we know whether its opening be in the middle, above, below, or at either side; we want, therefore, an instrument which will enable us to direct at will, the point of the bougie to the middle, upper, lower, or lateral part, in order that it may correspond with the aperture in the obstacle, and enter it.

To effect this, I make use of an instrument which I call a *conductor*. It consists of a gum-elastic catheter, of the size, No. 8 or 9, eight inches in length, open at both ends, and marked, like all my other instruments, with the divisions of the foot. I close the anterior extremity of this instrument, with a stopper of wax and silk, in order that the fluids in the urethra may not penetrate the interior of the conductor, and I mould this waxen stopper on the end of the catheter, so as to give it an uniformly rounded extremity. The instrument being oiled, is introduced down to the stricture, and the stopper is withdrawn. When the orifice of the stricture is in the centre, the canal forms, at the strictured part, a section of a cone, having at its apex the aperture through which the bougie is to pass. Now, my conductor also represents a section of a cone, open at its top, and the consequence is, that when the conductor is applied against the obstacle, its opening corresponds with that of the latter; so that a bougie necessarily enters the orifice of the stricture so soon as it has passed that of the conductor. To this we may add, that the bougie cannot vacillate in the conductor, and you may be certain that the introduction of a bougie by this method, is always very easy, when the opening in the stricture is at the centre.

When this opening is situated above, below, or at either side, the canal still represents in the strictured part, the section of a cone, but the opening is not at its summit; accordingly the opening of the conductor will not correspond with it, but with some solid part; the conductor, therefore, which I have just described, would not answer in this case. I then employ a conductor which has an eminence of a certain size at one of its sides, near its extremity. By this arrangement the orifice of the conductor is no longer in the middle of the instrument, but on one side. Having ascertained by the form of the impression, that the aperture in the stricture is situated at its upper part, I introduce the conductor, and turn downwards the eminence which it has at its extremity; the opening is thus turned upwards, and corresponds exactly with that of the obstacle, so that a bougie passing from the one, necessarily enters the other. If, on the contrary, the opening in the stricture be at the lower part, I direct the eminence in the conductor upwards; I turn it to the right when the aperture is at the left, and to the left when it is at the right; so that I direct at will the point of the bougie into the orifice of the stricture. We here perceive how just is the observation quoted by the learned and scientific reporter of the Royal Academy of Sciences, '*to be able to explore is a great part of the art*;' for the method of exploration above described, has reduced the introduction of bougies, formerly so uncertain and hazardous an operation, to a mathematical certainty." 110.

The bougie must be proportioned to the calibre of the stricture, and force must never be employed. M. Ducamp avers that by this method he always succeeds.

"I usually employ bougies which are not much more than eighteen lines in length, and I fasten them strongly on a tube of gum-elastic *Taille en biseau*, with waxed silk with which I form a bulb, that rests against the anterior opening of the conductor, which is less wide than its canal. On this tube of gum-elastic, or *porte-bougie*, I make two marks; one, which is at the entrance of the conductor when the bougie is about to pass out of it at the other end, indicates that the point of the instrument is in contact with the stricture; the other mark, about eighteen lines distant from the former, shows that the bougie has entered. When I wish to enlarge a stricture, I introduce, in the first place, a bougie with a well rounded point, or with one perhaps a little larger than the body of the instrument, in order that the latter may encounter no more resistance after the former has passed the obstacle. I immediately withdraw this bougie, and substitute another of similar dimensions as far as its middle; thence it increases in size to its other extremity. During the introduction this bougie passes easily, as far as its centre; when we have arrived at this point, resistance is felt; we may then press without fear of making a false passage, for the point of the bougie has traversed the obstacle, and the resistance met with is exercised upon the sides of the bougie, which distend the parietes of the stricture. I next secure the bougie and the conductor, and suffer them to remain in the urethra for half an hour; and if it appear necessary in the progress of the treatment, I repeat the operation the following day." 112.

M. Ducamp can also ascertain the extent of the stricture by means of another instrument, but we cannot stop to describe it. The preliminary knowledge necessary for proceeding to the destruction of the stricture having been acquired by these instruments and manœuvres, we are next presented with our author's means of arriving at that desirable result. It is by means of what he terms a *porte-caustic*. We are sorry to be necessitated to resort to so many extracts, but the nature of the descriptions renders it utterly impossible for us to abbreviate them more than we are doing.

"This instrument consists of a very pliable canula of gum-elastic, of the size No. 7 or 8, eight inches in length, (Pl. III. fig. 2.) and of a platina case or sheath eleven lines long, and of the same calibre as the gum-elastic tube. (Pl. III. fig. 3.) This sheath has *externally* one turn of a screw, by means of which it may be fitted to the gum-elastic tube, and be made continuous with it; at its other extremity is another screw, two lines and a half in length, on which is placed a little nut, (Pl. III. fig. 16.) rounded at its anterior extremity, and perforated in the middle to afford a passage for the central shaft of the instrument. The interior of the sheath presents, through one half of its circumference, two projecting ridges, which extend to its extremity, leaving between them on each side, and at two points diametrically opposite, a vacant space, which forms a groove from top to bottom. A cylinder of platina, ten lines long and one in diameter, attached to a gum-elastic bougie eight inches and a half in length, which serves as a handle to it, (Pl. III. fig. 5.) completes the instrument. This cylinder of platina has, five lines from its anterior extremity, a pin which extends beyond it for a quarter of a line right and left. At the distance of half a line below this pin, there is a deep furrow three lines in length and nearly three quarters of a line in breadth; thus prepared, the shaft represented in Plate III fig. 5, being introduced into the gum-elastic canula, and the platina sheath being firmly screwed on the tube, the extremity of the cylinder *b*, fig. 5, extends a little beyond that of the sheath *e*, fig. 4; and we have the instrument as represented by fig. 6. But if the shaft be pressed, the little cylinder of platina leaves the sheath, and the instrument appears as represented in Pl. III. fig. 7. When the groove *ff*, fig. 7, designed to contain the caustic, appears externally, its superior extremity is even with, and even somewhat covered by the sheath of platina, which terminates the gum-elastic tube. The platina nut being three lines in diameter, cannot enter the stricture, but rests upon its anterior surface, while the little cylinder of platina enters the opening of the same stricture, and applies to it three lines of caustic, which is directed at will, above, below, or laterally, and by means of which, consequently, we may cauterize a single point of the circumference, a greater or less extent of it, or even the whole, by giving certain motions to the instrument. Let us illustrate it by examples." 114.

The manner in which the caustic is directed to the stricture in different situations is accurately illustrated by examples, but we have not space for their insertion. M. Ducamp avers that its effects are really astonishing. A second, or at farthest a third, application is sufficient, in the majority of cases, to enable the patient to pass his urine with perfect freedom. After the first application M. Ducamp waits for three days without doing any thing. He then takes a new impression, to ascertain the state of the parts; passes a bougie by which he learns if there be but one stricture, and then repeats the application of the caustic, directing it to the most prominent parts. In three days more a new impression is taken, and if the stricture projects little, a bougie, No. 6, may be passed with ease. Unless the stricture still projects considerably he makes no farther application of the caustic, but proceeds to dilate the canal by means to be yet described. A second and a third stricture are successively attacked in a similar manner. If the stricture be deeper in the canal than six inches, the instrument must be slightly modified to accommodate it to the urethral curve. The extent of the application of the caustic must be proportioned to that of the stricture, but if the latter should be very long it is better to destroy it progressively by different applications of two or three lines each. The quantity of caustic expended each time should not exceed the tenth of a grain; the less caustic possible for the destruction of the stricture the better. The groove in the porte-caustic is filled with the nitrate of silver, by placing two little bits of it in the groove, and melting these carefully in the flame of a spirit lamp; if the caustic does not lie evenly, it must be levelled by a piece of pumice-stone. The groove holds nearly half a grain of caustic, but by suffering the instrument to remain for a minute only, not more than a third is dissolved. No force must be used with this instrument, nor must caustic be used whilst the canal is in a state of inflammation. Having proceeded thus far, the object that remains is to effect an adequate dilatation of the urethra, and, as M. Ducamp words it, "to obtain a CICATRIX of the calibre of the urethra in its natural state." For this purpose he employs two instruments, a *dilator* and a *bellied bougie*. It will be remembered that Mr. Arnott has proposed as a dilator, a catgut bag which he fills with air. The original proposal is in the works of Desault. Now the dilators of M. Ducamp are on this principle.

"I usually employ three dilators: the first is three lines in diameter, the second nearly four, and the third four and a half. I make the first and second of the appendix vermiformis of the cæcum, and the third, with a bit of catgut; and, causing them to be prepared by a manufacturer of catgut, I measure them with a compass. Some of them are three lines in diameter at the apex, and there are others which, particularly at the base, are four lines across. I cover a little silver rod, which terminates in a bulb, with a piece of the appendix about twenty lines in length; the sack, formed by the intestines, is tightly drawn over the bulb of the rod, and fastened under it with silk, tied in a double knot. This being done, I pass the rod into a canula of the same metal, eight or nine inches in length, having, at its anterior extremity, a deep groove three lines long, and I fasten the loose end of the appendix to this groove, securely, with waxed silk. (Pl. IV., figs. 1 and 4.) The silver canula has at its other extremity a cap or button, furnished with one turn of a screw. The rod of silver above-mentioned, must project a little beyond this cap, (Pl. IV. fig. 2,) and not fill the cavity of the canula exactly; it should play freely within it, and on pressing the end to which the dilator is attached, the other ought to pass out beyond the cap. Thus, we have an instrument which, for the space of eighteen lines, is not longer than the second size of bougies, when empty, and acquires a diameter of three lines when full. (Pl. IV. fig. 1. and explan).

It is seldom that we can meet with a vermicular appendix four lines in diameter at the apex, though they are frequently of this size at the base. I take a piece of the intestine of this calibre, to make the second dilator; and as this does not terminate in a sack, like that employed for the first, I fasten it strongly with silk, for the space of three lines, to the end of the silver rod. After this, I turn back the piece of appendix upon the ligature just mentioned, and finish the second dilator like the first. The third kind is similarly prepared, but with a piece of catgut four lines and a half in diameter. (Pl. IV. figs. 2 and 3.)

These instruments are to be used as follows:—Mark upon the canula, with a little wax, the distance from the external orifice to the stricture, so that the middle of the sack of the dilator may correspond with the part to be distended, when the mark shall be at the orifice in the glans. The dilator, being moistened and dipped in oil, is to be introduced like a catheter. If the extremity of the dilator meet with any resistance, by which its progress is impeded, it will be immediately perceived by the silver shaft passing out beyond the cap. We must not then press with our fingers, or in any other manner, but withdraw the instrument, and change its direction. I always introduce my dilators without either pressing the rod, or fastening it so that it cannot slip back. I believe this mode of proceeding to be prudent, and I should consider as very reprehensible any other, having for its object the forcible introduction of these instruments. I again repeat it, and I think it cannot be too often reiterated, that the forcible introduction of instruments, in the treatment of strictures in the urethra, can never be other than pernicious.

When the dilator is introduced, I fit, by means of a screw, a syringe (Pl. IV. fig. 6,) furnished with a fawcet to the cap of the dilator, and I press on the piston gently until I meet with some resistance; I then turn the fawcet; the dilator is now distended, and separates the parietes of the strictured portion from three to four lines. In about five minutes, for the first time, and afterwards from ten to fifteen, I open the fawcet, empty the instrument, and withdraw it.

For a long time I distended my dilators with air: I used then to have a good deal of trouble to make the dilatation permanent; for it is very difficult, even with the greatest care, to prevent the air from escaping through some outlet. When this fluid is strongly compressed, it finds its way between the fawcet and the little circular piece of leather which separates them. I have remedied this inconvenience, by filling the instrument in the following manner: I inject air into the dilator until I experience a slight resistance, and turn the fawcet; I fill the syringe, and attaching it to the fawcet, the latter is opened, and the water thrown in upon the air; the latter, strongly compressed by the column of water, becomes condensed; the quantity of air which occupied the whole dilator, no longer fills more than half or one-third of it, the rest being occupied by the water. In this way, a much more powerful distention is produced, and if the dilator should leak, the compressed air would expand, and, supplying the deficiency, keep up the dilatation. Besides, it is then impossible for the air to escape through the fawcet and tent; for between these parts of the instrument and the place occupied by the air, is a column of water which the former cannot pass. In this manner the dilator is distended to its fullest extent, and may be kept in that state for a longer or shorter time. Hence, on opening the fawcet after a quarter of an hour, the air, recovering its natural density, throws to a distance a part of the water contained in the instrument—a proof that the latter was powerfully distended." 123.

The distention produced by this instrument not being permanent, M. Ducamp assists it by what he calls the *bellied bougie*. This is a bougie made with a protuberance or belly, twelve to fifteen lines in extent. He has bougies with bellies of different dimensions, the shaft being in the smallest, as in the largest, only two lines in diameter. The smallest, which he uses immediately after having destroyed the stricture with caustic, has a belly two lines and a half in diameter; the largest has a belly four lines in diameter. The advantages are said to be, 1, their introduction is less difficult and not so painful as that of the other kinds; 2, they distend only the part strictured; 3, they can distend to the degree of four lines, while the others do not dilate it above three lines.

"To resume the course of our treatment: three days after the last application of the nitrate of silver, I introduce a dilator three lines in diameter, inflated with air, and suffer it to remain five minutes; the next day I introduce the same dilator, distended as much as possible with air and water; in about ten minutes, I remove it, and put in its place a bellied bougie, two lines and a half in diameter, which the patient keeps there twenty minutes: the introduction of this bougie is repeated the next day, both morning and evening, and suffered to remain a similar time. The day after, I pass the second dilator, of nearly four lines diameter, and withdrawing it after ten minutes, its place is supplied by a bellied bougie three lines in diameter. This bougie is passed, morning and evening the next day, remaining in, each time, from ten to twenty minutes. A further dilatation is effected the next day with the same dilator; two days after, the third dilator is introduced, having a diameter of four lines and a half, and succeeded by a bellied bougie of three lines and a half. After another interval of two days, I again introduce this dilator, and pass a bellied bougie four lines in diameter, which is repeated morning and evening, remaining a quarter of an hour. In the course of a week, the bougie is only introduced once a day, and retained but for a few minutes: in about four or five days, the patient himself passes it once a day, withdrawing it immediately.* The cicatrix, by this time, has become firmly consolidated, and is four lines in diameter, like the rest of the canal." 130.

Such is M. Ducamp's treatment of ordinary strictures; and it now only remains for us to mention his method in cases of complete retention.

---

* "When we have arrived at this stage, it is desirable that the belly of the bougie should not be more than four or five lines in length, (Pl. IV. fig. 8) so that the friction being less considerable, the introduction may be rendered easier."

"I take a fine gum-elastic bougie, and introduce it gently into the urethra; if I succeed in making it pass through the obstacle, I suffer it to remain until a strong inclination to make water is felt, and then withdraw the instrument softly, and the urine rushes through the passage which had been occupied by the instrument, flowing in a stream. When the patient has voided as much urine as possible, I introduce the bougie again, and leave it until a fresh inclination to make water comes on. In the mean while I bleed the patient, and apply twenty or thirty leeches to the anus and perineum, and prescribe a hip-bath, emollient anodyne clysters, rest, and abstinence.

If I cannot pass a bougie I use no force to thrust it in, but remove it. I take an impression of the stricture, and introduce a small bougie by means of a conductor; this bougie is succeeded by one of greater size; when the inclination to make water is very urgent, I withdraw at once both bougie and conductor, and the stream flows. I have recourse afterwards to the above-mentioned antiphlogistic means, and when the inflammation of the canal has been subdued, I commence the radical treatment. This mode of proceeding has always succeeded with me, and I have never adopted any other since I have entertained the ideas suggested in this chapter. If, which is not probable, the bougie should fail to re-establish the course of urine, it will be necessary, after having passed the second bougie, to introduce, through a conductor, a small gum-elastic catheter into the bladder, and draw off the urine in this manner." 133.

Such is the new method of treating stricture proposed, nay employed, by M. Ducamp. We doubt if it will ever find its way into general favour and use. It is too complex, too troublesome, to suit the generality of surgeons. The advantages attributed to it by its inventor may be said, in a general sense, to be two—the speedy and easy destruction and dilatation of the stricture, and the permanency of the cure. With regard to the first, many surgeons will probably think that the ordinary treatment by bougies is, in the main, both simple and efficacious in effecting dilatation. We must say that, from what we have seen and from our own experience, we feel no inclination to abandon the use of the bougie, that is, of our ordinary methods of dilatation; and experience is rather against than for the employment of caustic. If bougies—and when we use this term, we mean the treatment by dilatation generally, employing, according to circumstances, bougies, gum and metallic catheters—if bougies, then, be carefully and dexterously used by a light hand and a practised one, the surgeon is seldom, very seldom foiled, even in cases of the worst description. The treatment by dilatation is more safe, and equally or more speedy than that by caustic, though the latter is now and then extremely serviceable.

But the ground on which M. Ducamp seems mainly to lean, is that of the permanency of the cure by his method. What guarantee have we of that permanency? on what known principle should dilatation, effected by caustic, be thus permanent? We know of none that can warrant the assumption—nay, facts and experience contradict it. We shall not commence a disputation on the subject, as the reasons for our opinion will readily present themselves to the minds and memories of medical men. Our space has been so occupied by placing the propositions of our author before our readers, that really we have none for discussions or arguments. We leave these propositions in the hands of our professional brethren, to believe, disbelieve, put them to the test of experience, or pass them by in silence, as they think most proper.

